# Efficacy and safety of switching from febuxostat to dotinurad, a novel selective urate reabsorption inhibitor, in hyperuricemic patients with type 2 diabetic kidney disease: Protocol for a single-arm, open-label, prospective, exploratory study

**DOI:** 10.3389/fendo.2022.1042061

**Published:** 2023-01-11

**Authors:** Takeshi Osonoi, Miyoko Saito, Mitsuru Hosoya, Satako Douguchi, Kensuke Ofuchi, Makoto Katoh

**Affiliations:** Naka Kinen Clinic, Ibaraki, Japan

**Keywords:** febuxostat, dotinurad, type 2 diabetic kidney disease, urate excretion transporter, serum urate, protocol, indoxyl sulfate

## Abstract

**Background:**

Dotinurad is a novel uricosuric drug in Japan with selective and potent urate transporter 1 (URAT1) inhibitory activity. This study aims to evaluate the efficacy and safety of dotinurad in hyperuricemic patients with type 2 diabetic kidney disease by comparing serum levels of urate and plasma and urinary levels of indoxyl sulfate excreted *via* the urate excretion transporter ATP binding cassette subfamily G member 2 (ABCG2), as indices, with baseline levels after switching from febuxostat to dotinurad.

**Methods:**

This single-center, single-arm, open-label, prospective, exploratory study aims to evaluate the effect of switching from febuxostat to dotinurad on serum urate levels and its background factors. The study will include 50 hyperuricemic patients with type 2 diabetic kidney disease and urate levels exceeding 6 mg/dL despite administration of febuxostat 20 mg/day for at least 3 months. The primary outcome is the achievement rate of serum urate levels of ≤6 mg/dL after 24 weeks of treatment with dotinurad at 0.5 mg to a maximum of 4 mg once daily. Secondary outcomes include the changes in serum urate levels, plasma and urinary indoxyl sulfate levels, and renal injury-related markers from baseline to observation points at weeks 4, 12, and 24.

**Discussion:**

The study hypothesizes that switching to dotinurad may reduce the plasma levels of indoxyl sulfate and increase its urinary levels in patients with hyperuricemia. These suggest that dotinurad can potently lower the serum urate level by inhibiting URAT1 without adversely affecting ABCG2. Thus, findings of this study are expected to provide useful insights into the treatment of hyperuricemia associated with type 2 diabetic kidney disease and the discovery of new possibilities for dotinurad.

**Ethics and Dissemination:**

Prior to the study, its study protocol was scientifically and ethically reviewed and approved by the Japan Physicians Association Clinical Research Review Board (approval number: JPA007-2204-02). In addition, patients who provide written informed consent will participate in the study. The results of this study will be published through submission to a peer-reviewed scientific journal.

**Clinical trial registration:**

https://jrct.niph.go.jp/en-latest-detail/jRCTs031220080, identifier jRCTs031220080.

## 1 Introduction

The incidence of gout is increasing every year in Japan, with an estimated 1.1 million patients according to the Comprehensive Survey of Living Conditions, while the number of patients with hyperuricemia is approximately 10 million or more (1). In recent years, it has been shown that hyperuricemia not only causes gout but is also closely related to lifestyle-related diseases, such as hypertension, type 2 diabetes, and atherosclerosis, even in asymptomatic patients and may be associated with the onset and progression of chronic kidney disease (CKD) (2–4). In particular, type 2 diabetes is often complicated by hyperuricemia, and patients with these diseases have a significantly increased risk of developing CKD compared with those without hyperuricemia (5). Furthermore, high serum urate levels are a possible risk factor for CKD progression in patients with type 2 diabetic kidney disease; however, there are few reports on this possibility.

Hyperuricemia is classified into reduced urate excretion, renal overload (urate overproduction and reduced extra-renal excretion), and mixed types. The prevalence of hyperuricemia is approximately 60% for the reduced excretion type, 30% for the mixed type, and 10% for the renal overload type; hyperuricemia with reduced excretion features accounts for the majority of cases (1). This is likely due in part to the progression of CKD and a decreased glomerular filtration rate (GFR): an increase in urate levels with a decrease in urate excretion. Currently, urate-lowering drugs include xanthine oxidoreductase (XOR) inhibitors, which inhibit urate production, and uricosuric drugs; however, XOR inhibitors are recommended for patients with renal impairment, regardless of the type of hyperuricemia (1, 6).

Urate excretion transporters include URAT1, located in the proximal tubules of the kidney and is responsible for urate reabsorption, and ABCG2 and organic anion transporter 1 (OAT1) and OAT3, which are involved in excretion (7, 8). ABCG2 exits in the kidney and the intestinal tract and secretes urate (9). These urate excretion transporters are involved in the renal excretion of both urate and indoxyl sulfate, a uremic substance (10). Uremic substances accumulate in the blood and various organs and are risk factors for cardiovascular complications and other problems as well as progression to CKD (11). Furthermore, the XOR inhibitor febuxostat and the uricosuric drug benzbromarone inhibit ABCG2-mediated urate transport (12); in a previous study, febuxostat or benzbromarone increased the plasma levels of ABCG2-probe substance in rats (13). Therefore, in patients with hyperuricemia complicated by renal impairment, urate-lowering drugs that do not affect urate excretion transporters are expected to provide potent urate reduction and to reduce the progression of renal disease and the development of cardiovascular disease.

Dotinurad is a novel selective urate reabsorption inhibitor (SURI) that selectively and potently inhibits URAT1 but with little effect on ABCG2, OAT1, or OAT3 (14). However, to the best of our knowledge, the efficacy and safety of switching from the XOR inhibitor febuxostat to dotinurad in hyperuricemic patients under clinical conditions have not yet been investigated.

## 2 Methods and analysis

### 2.1 Design

The SWITCH SURI Study (efficacy and safety of SWITCHing from febuxostat to dotinurad, a novel Selective Urate Reabsorption Inhibitor, in hyperuricemic patients with type 2 diabetic kidney disease) is a single-center, single-arm, open-label, prospective trial. This is an exploratory study aimed at evaluating the effect of switching from febuxostat to dotinurad on the serum urate levels and renal impairment parameters, as well as the underlying factors and to evaluate the incidence of adverse events and changes in safety measures, such as liver enzymes, glucose, and metabolism (**Table 1**). This study will evaluate the rate of achievement of serum urate levels to ≤6 mg/dL at week 24 after switching to dotinurad.

**Table 1:** Overview of All Visits and Tests Schedule.

Item	At informed consent	Observation period				At discontinuation
		Week 0	Week 4	Week 12	Week 24	
Permitted ranges of visits		0	±2 weeks	±4 weeks	±4 weeks	–
Informed consent	✓					
Demography	✓					
Study drug adherence			✓	✓	✓	✓
Concomitant therapy status		✓	✓	✓	✓	✓
Height, body weight		✓	✓	✓	✓	✓
Blood pressure, pulse rate		✓	✓	✓	✓	✓
Hematology tests		✓	✓	✓	✓	✓
Urinalysis		✓	✓	✓	✓	✓
Adverse events			✓	✓	✓	✓

Dotinurad will be started with Urece^®^ 0.5 mg tablet orally once daily within 1 week after the end of the baseline examination, and if the urate level remains above 6 mg/dL after ≥2 weeks, the dose of Urece^®^ tablets will be gradually increased (up to 4 mg), at observation points and at other scheduled visits, and continued until week 24 of the observation period.

Because of the potential to affect the evaluation of this study, the concomitant use of urate-lowering drugs (other than dotinurad), drugs that act on urate transporters (sodium-glucose cotransporter 2 [SGLT2] inhibitors, losartan, irbesartan), or drugs that affect indoxyl sulfate levels (spherical adsorptive carbon) is prohibited until the patient visit at 24 weeks after the start of dotinurad administration (except in case of discontinuation). However, in principle, drugs that patients already take before they provide consent may be continued from the date of consent until week 24, and the dosage and administration should be increased or decreased as appropriate according to medical conditions. Moreover, drugs deemed medically necessary may be added, except for the above prohibited concomitant medications.

### 2.2 Sample selection

Patients with type 2 diabetic kidney disease and hyperuricemia with urate levels exceeding 6 mg/dL even after receiving febuxostat 20 mg/day for at least 3 months who meet inclusion criteria and do not conflict with exclusion criteria will be eligible.

#### 2.2.1 Inclusion criteria

1. Patients with serum urate levels greater than 6.0 mg/dL and less than 10 mg/dL within 8 weeks before the day of consent
2. Patients with type 2 diabetes mellitus
3. Patients on febuxostat 20 mg/day for at least 3 months who have not changed their diabetes medication dosage from at least 8 weeks before the day of consent until the day of consent
4. Patients with renal impairment within 8 weeks before the date of consent and renal function between 30 and 60 by eGFR (mL/min/1.73 m^2^)
5. Patients with eGFR measurement data available from 6 months ( ± 2 months) before the day of consent
6. Patients aged ≥20 years on the day of informed consent (either sex)
7. Patients who have provided their written informed consent to participate in this study.

#### 2.2.2 Exclusion criteria

1. Patients with severe renal impairment (eGFR of <30 mL/min/1.73 m^2^) or end-stage renal failure on dialysis
2. Patients with unsettled post-acute gouty arthritis
3. Patients with a history of any of the following diseases within 12 weeks before the date of consent: acute coronary syndrome, cerebrovascular disease, myocarditis, constrictive pericarditis, or severe valvular disease
4. Patients with hepatic impairment (aspartate aminotransferase [AST] or alanine aminotransferase [ALT] of ≥100 IU/L)
5. Patients with a diagnosis or history of urinary tract stones
6. Patients diagnosed with malignancy or suspected or suspected of having malignancy (however, if the physician determines that the patient has not been treated with anticancer drugs for one year before the date of consent and does not plan to do so in the future, the patient is eligible)
7. Female patients who are pregnant, lactating, or may be pregnant
8. Patients receiving uricosuric drugs, SGLT2 inhibitors, losartan, irbesartan, or spherical adsorptive carbon within 8 weeks before the day of consent
9. Patients requiring a surrogate
10. Patients with a history of hypersensitivity to any ingredients of the dotinurad product
11. Other patients who are judged by the investigator to be inappropriate for this study

After patient selection, the principal investigator or the subinvestigator will explain to the patients the details of the study using a consent explanation document approved by an accredited clinical research review committee and obtain their written consent. The principal investigator or the subinvestigator will also explain that consent is given freely by the study participant and that the subject will not be treated unfavorably even if he/she does not consent, and that the participant may withdraw consent at any time after consent is given if he/she changes his/her mind, and that the subject will not be treated unfavorably in such cases.

### 2.3 Outcome assessment

#### 2.3.1 Primary outcome

The primary outcome of this study will be the rate of achievement of serum urate levels of ≤6 mg/dL at week 24 of dotinurad treatment.

#### 2.3.2 Secondary outcomes

The secondary outcomes of this study will include the following from baseline to observation points at weeks 4, 12, and 24.

1. Change in serum urate levels
2. Changes in plasma indoxyl sulfate
3. Renal impairment-related parameters: changes in eGFR, eGFR slope, serum creatinine, and urinary albumin and creatine (urine will be collected at any time and levels corrected for urinary creatine will be used)
4. Changes in doses of the study drug used in the study

#### 2.3.3 Safety outcomes

The safety outcomes will include the following items.

1. Incidence of adverse events and diseases
2. Liver function markers: changes in AST, ALT, and γ-glutamyl transpeptidase (γ-GTP)
3. Glucose and metabolic markers: changes in hemoglobin A1c (HbA1c) and body mass index (BMI)
4. Blood pressure and pulse: changes in systolic and diastolic blood pressure and pulse rate
5. Electrolytes: changes in serum sodium and urinary sodium

#### 2.3.4 Exploratory outcomes

The exploratory outcomes will include the following items.

1. Changes in urinary levels of urate and indoxyl sulfate
2. Inflammation oxidative stress markets: changes in serum high sensitive C-reactive protein (hs-CRP) and in urinary 8-hydroxydeoxyguanosine (8-OHdG)
3. Comparison between groups of patients who achieved and did not achieve serum urate levels of ≤6 mg/dL (baseline levels and changes)

Two analysis groups will be set for the efficacy of this study: Full analysis set (FAS) and per-protocol set (PPS), with FAS being the primary analysis. The FAS will be the population of eligible patients who have received the study drug and have serum urate readings at baseline and at least one time point during the observation period. The PPS will be the population from the FAS excluding the participants who deviated from the study protocol (e.g., use of concomitantly prohibited drugs or study drug adherence of <80%). The Safety Analysis Set (SAS) analysis population will be established for the safety of the study, and the SAS will be the population in which the study drug is administered and data on any of the safety outcomes are obtained.

### 2.4 Sample size calculation

The target sample size will be set at 50. To evaluate the efficacy of switching to dotinurad in the participants in the study, a threshold value of 30% was set based on the achievement rate of serum urate levels of ≤6 mg/dL (20%) with 40 mg of febuxostat. To demonstrate that the lower limit of the two-sided 95% confidence interval exceeds the threshold of 30%, a significance level of α = 0.05 (two-sided) and a power of 1-β 90%, the required sample size was calculated by a binomial probability hypothesis test, resulting in 44. Considering dropouts, the target sample size will be set at 50.

### 2.5 Statistical analysis

Summary statistics of the background data of the study participants will be calculated. For nominal variables, the numbers in each category and their percentages will be shown; for continuous variables, the numbers, mean, standard deviation, minimum, median, and maximum values will be calculated.

The two-sided 95% confidence interval for the primary efficacy outcome of achieving a serum urate level of ≤6 mg/dL at week 24 of dotinurad treatment will be calculated, and a one-sample population proportion test will be performed for the null hypothesis of an achievement rate equal to 30%. Subgroup analysis will be performed by other candidate factors, such as subject background, to examine their impact on the primary outcome. For the secondary outcomes obtained from the metric data, summary statistics will be calculated at each observation point, a one-sample t-test or Wilcoxon’s signed rank test will be performed to evaluate their statistical significance, and if necessary, analysis of covariance and multivariate analysis will be performed. If the data are non-normally distributed, appropriate data transformations will be applied. When statistical tests are conducted, the significance level will be set at 0.05 two-sided, with the confidence coefficient for statistical estimation being 95% two-sided. Missing data in efficacy will be imputed using the last observation carried forward method.

## 3 Discussion

The efficacy of switching from febuxostat to dotinurad in patients with hyperuricemia or dotinurad in patients with type 2 diabetic kidney disease has not yet been studied in clinical setting. Furthermore, switching to dotinurad may affect plasma and urinary indoxyl sulfate levels in hyperuricemic patients, suggesting that dotinurad can potently lower the serum urate level by inhibiting URAT1 without adversely affecting ABCG2. Thus, the results of this investigation in this study may provide new insights into strategies for treating hyperuricemia with dotinurad.

It is known that URAT1 inhibitors may be associated with high incidence of serum creatinine elevations and renal-related adverse events (15). The mechanism underlying the elevation of sCr levels is not fully known, but it may be due to increased excretion and microcrystallization of urinary uric acid in renal tubules. A long-term phase 3 study assessing the safety of dotinurad monotherapy demonstrated no noteworthy changes with respect to renal impairment and renal parameters (16). In addition, renal function had significantly improved from the baseline with long-term treatment on dotinurad (16). Hence, the risk of developing renal-related adverse events by dotinurad in this study is considered low.

The dose of febuxostat was selected based on the highest percentage of the dose (20 mg/day) prescribed in real-world clinical practice. In other words, the maintenance dose of febuxostat for hyperuricemia is usually 40 mg/day, but in a report (17) using the JMDC Epidemiology Receipt Database in Japan, the percentage of febuxostat doses prescribed among 6,118 patients was 40.9% for 10 mg/day, 49.9% for 20 mg/day, 8.7% for 40 mg/day, 0.5% for 60 mg/day, indicating that lower doses than the maintenance dose were most commonly prescribed. Furthermore, the cumulative achievement rates of febuxostat for serum urate levels of ≤6 mg/dL in patients with gout or asymptomatic hyperuricemia (17) or with hyperuricemia and CKD (18) were 51% and 41% at 20 mg/day and 60% and 53% at 40 mg/day, respectively, with no significant increase in the efficacy for an increase to the maintenance dose. In addition, febuxostat may be hesitant to increase the dose because the CARES trial, received at an initial dose of 40 mg/day febuxostat, showed a higher incidence of CV events than allopurinol (19). Thus, providing better treatment for patients who do not achieve serum urate levels of ≤6 mg/dL during the treatment of hyperuricemia with febuxostat may avoid clinical inertia.

**Figure 1:**
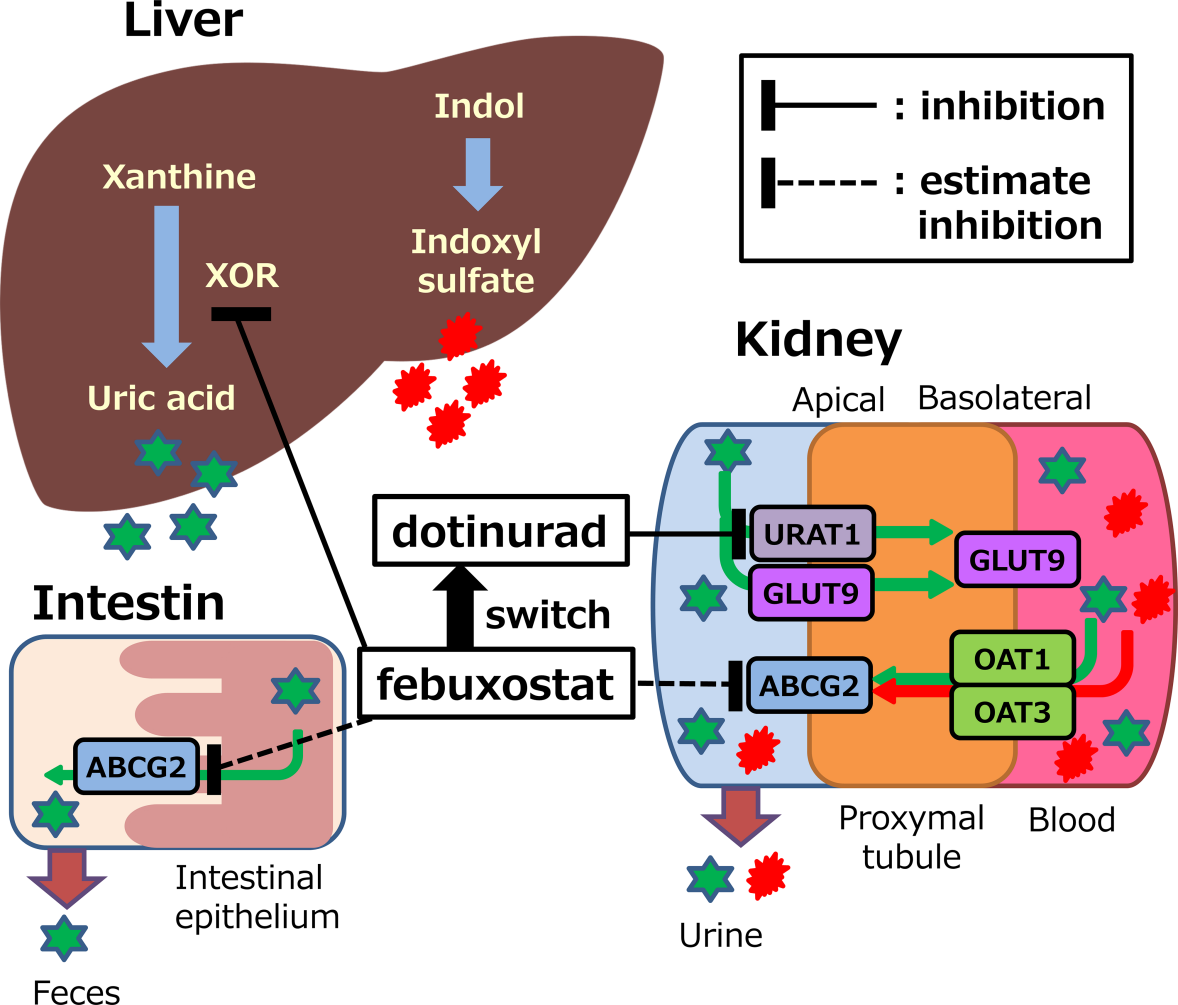
Potential mechanisms of action of febuxostat or dotinurad on synthesis and excretion of uric acid and indoxyl sulfate. XOR, xanthine oxidoreductase; URAT1, urate transporter 1; ABCG2, ATP binding cassette, subfamily G, 2; OAT, organic anion transporter; GLUT9, glucose transporter 9.

Nonclinical studies reported that febuxostat inhibits the urate excretion transporters ABCG2. OAT1, and OAT3 (12, 13). These urate excretion transporters are involved in the renal excretion of both urate and indoxyl sulfate (10). Thus, febuxostat may inhibit the excretion of urate and indoxyl sulfate (**Figure 1**). In contrast, dotinurad exerts a selective and potent URAT1 inhibitory activity with little effect on ABCG2, OAT1, or OAT3 (14). However, indirect or direct evaluation in clinical setting has not been studied yet.

In this study, indoxyl sulfate will be used as an indicator of effects on ABCG2, OAT1, and OAT3. In patients with impaired renal impairment, the level of indoxyl sulfate in the circulating blood increases, thus a risk factor for CKD progression and cardiovascular complications due to its accumulation in the blood and various organs (11). Thus, lowering plasma indoxyl sulfate concentrations with dotinurad may be associated with a lower risk of cardiovascular disease development and renal disease progression than febuxostat. Therefore, we will also evaluate the eGFR slope before and after dotinurad treatment and its effect on renal function decline. However, further studies are needed for the development of cardiovascular disease, which requires long-term observation periods in large clinical studies.

In a recent prospective, randomized, open-label study comparing the effects of the urate excretion-promoting agent benzbromarone 25 mg/day with febuxostat 20 mg/day in male patients with gout with reduced urinary urate excretion type, the rate of achieving a serum urate target of <6 mg/dL after 12 weeks of treatment was higher in the benzbromarone group than in the febuxostat group (20). Benzbromarone also inhibits urate transport *via* ABCG2, as does febuxostat (12), suggesting that URAT1 inhibitors are more potent in lowering serum urate than XOR inhibitors in hyperuricemia of reduced urate excretion type. Because patients receiving febuxostat will be enrolled in this study, it is not possible to confirm the type of hyperuricemia; however, in general, the majority of patients have a reduced urate excretion type (1). Thus, dotinurad may be more potent than febuxostat in lowering serum urate through its action *via* URAT1 inhibition alone.

Urate is synthesized primarily in the liver, approximately 70% of which is excreted by the kidneys and the remaining 30% by the small intestine (21, 22). In particular, urate excreted from the small intestine is mediated by ABCG2 (9, 23). Thus, dotinurad can be considered to hardly inhibit ABCG2-mediated urate excretion, and the potential to excrete urate from two organs, the kidneys and the small intestine. This may be related to the very high rates of achievement of serum urate levels of <6 mg/dL (91.3% at 2 mg/day and 100% at 4 mg/day) observed in an open-label phase 3 study of dotinurad treatment for 58 weeks (16).

Although this study has the limitation of a short observation period to evaluate events and is an open, single-arm study on a small scale, it is the first study that switching to dotinurad may reduce the plasma levels of indoxyl sulfate and increase its urinary levels, which may be an indication of the effect on ABCG2, OAT1, and OAT3. In addition, there is a critical issue that the proportion of women has been underrepresented in clinical trials testing urate-lowering drugs (24). This study is being conducted on consecutive eligible patients without consideration of gender. Therefore, it is unlikely to be underrepresented compared to proportion of women in population suffering from asymptomatic hyperuricemia.

## 4 Trial status

The study protocol was fixed on April 1, 2022, and enrollment has started at the time of submission of this paper. Enrollment is expected to be completed by April 30, 2023.

## 5 Ethics and dissemination

Prior to the study, the study protocol was scientifically and ethically reviewed and approved by the Japan Physicians Association Clinical Research Review Board (approval number: JPA007-2204-02). The study protocol was registered in the Japan Registry of Clinical Trials (jRCT) in May 2022 (jRCTs031220080) and is now available. This study will be conducted at the Naka Kinen Clinic in accordance with the ethical principles of the Declaration of Helsinki and in compliance with the “Clinical Research Act.” The physician in charge of the study will provide sufficient explanation to the study participants using the consent and explanation documents and obtain their free and voluntary consent in writing. The results of this study will be published through submission to a peer-reviewed scientific journal.

## Data availability statement

The datasets presented in this study can be found in online repositories. The names of the repository/repositories and accession number(s) can be found below: https://jrct.niph.go.jp/en-latest-detail/jRCTs031220080.

## Ethics statement

The studies involving human participants were reviewed and approved by Japan Physicians Association Clinical Research Review Board (approval number: JPA007-2204-02). The patients/participants provided their written informed consent to participate in this study.

## Author contributions

TO and MK are involved in the planning of the study. All authors are involved in the study design. TO and MK are involved in drafting this article. All authors read and critically reviewed this manuscript. All authors contributed to the article and approved the submitted version.
